# Body Mass Trajectory Affects the Long-Term Occurrence of Metabolic Syndrome in Adult Patients with Severe Obesity

**DOI:** 10.3390/children10010027

**Published:** 2022-12-23

**Authors:** Juliette Hazart, Florence Montel, Elodie Gentes, Clément Lahaye, Mélanie Pouget, Nicolas Farigon, Magalie Miolanne, Aurélien Mulliez, Yves Boirie

**Affiliations:** 1 CHU Clermont-Ferrand, Service de Nutrition Clinique, CRNH Auvergne, Université Clermont Auvergne, 63000 Clermont-Ferrand, France; 2 INRA, Unité de Nutrition Humaine (UNH), CRNH Auvergne, Université Clermont Auvergne, 63000 Clermont-Ferrand, France; 3 Délégation Recherche Clinique & Innovation, CHU Clermont-Ferrand, 63000 Clermont-Ferrand, France

**Keywords:** childhood obesity, insulin resistance, metabolically healthy obesity

## Abstract

Independently of absolute BMI values, the amount, onset date, and duration of early body weight gain may influence cardio-metabolic health later in adulthood. Values of cardiac and metabolic variables from a cohort study of morbidly obese patients were retrospectively analyzed to study the association between early weight history and metabolic syndrome (MetS) occurrence in adults. Of 950 patients with severe morbid obesity (age 44.3 ± 13.8 y, BMI 42.5 ± 7.0 kg/m^2^), 31.4% had started excess weight gain in childhood (CH), 19.9% in adolescence (ADO), and 48.7% in adulthood (AD). Despite different BMI values, MetS prevalence (57.8%) was not significantly different in the three groups (54.4% CH vs. 57.7% ADO vs. 59.8% AD, *p* = 0.59). The overweight onset period was not significantly associated with the development of MetS in adults (ADO: OR = 1.14 [0.69–1.92], *p* = 0.60; AD: OR = 0.99 [0.62–1.56], *p* = 0.95) despite a higher BMI in the early obesity onset group. Weight gain of more than 50% after age 18 years significantly increased the risk of MetS (OR = 1.75 [1.07–2.88], *p* = 0.026). In addition to crude BMI values, analysis of body mass trajectories is a relevant clinical tool in the assessment of metabolic risk, suggesting that the magnitude of weight gain may be more important for metabolic syndrome progression than the period of obesity onset.

## 1. Introduction

According to the World Health Organization, overweight and obesity affected 36.9% of adult males, 38.0% of adult females, 12.9% of male children and teenagers, and 13.4% of female children and teenagers in developed countries in 2013 [1]. In 2015, a total of 107.7 million children and 603.7 million adults were obese. Although the prevalence of obesity among children has been lower than among adults, the rise in childhood obesity has outpaced adult obesity in many countries [2]. In France, the prevalence of overweight and obese children in 2015 was 16% in males and 18% in females, with no significant evolution since 2006 [3]. Obesity in adulthood is a well-known risk factor for morbidities, such as type 2 diabetes, dyslipidemia, and hypertension, which can co-occur in metabolic syndrome, insulin resistance, and all-cause mortality [4,5]. Nevertheless, some obese subjects unexpectedly keep a favorable metabolic profile, pointing to separate metabolically healthy and metabolically unhealthy obesity phenotypes [6,7]. The estimated prevalence of the metabolically healthy obese (MHO) phenotype ranges from 6 to 75%, according to definitions [8]. Numerous mechanisms could explain a more favorable metabolic profile, including an interaction between genetic and environmental factors, better oxidative capacities, less active systemic inflammation, lower development of visceral adipose tissue, or a healthy expansion of the adipose tissue overall [9,10]. The precocity of overweight history, and particularly the dynamic period of excessive adiposity development, may be crucial for future metabolic complications. During childhood, body tissues may possess higher metabolic adaptation capacities through greater adipose tissue cell plasticity together with smaller adipocyte size [11]. Obesity onset during childhood compared with obesity during adulthood might thus be characterized by a stronger adaptive capacity to cope with an excessive energy balance and enable improved storage capacity in adipose tissue. This better adaptation might thus limit exposure of peripheral tissues to ectopic fat deposition and reduce metabolic risk, leading to a paradoxical “less unfavorable” metabolic profile in adulthood despite a high BMI value. For instance, the favorable metabolic profile of a population of obese menopausal women was associated with the early development of obesity compared with obese subjects with metabolic abnormalities [12]. In another review, no evidence was found for an independent association between childhood obesity and metabolic syndrome in adulthood after adjusting for BMI [13]. It is suggested that rather than BMI during childhood, the magnitude of lifetime weight gain leading to obesity in adulthood may more strongly influence the association between childhood obesity and cardiovascular risks [14]. However, other authors found no potential “protective” cardiometabolic role of premature obesity or a rapid BMI increase in childhood [15]. The association between an individual’s body mass index history and their metabolic risk in adulthood is complex, and data in the current literature are still conflicting. The main objective of our study was to determine the long-term association between overweight history (in particular, the period of obesity onset), BMI and weight gain in adulthood and the according development of metabolic syndrome in adulthood in a population of severe (35 ≤ BMI ≤ 40 kg/m^2^) and morbidly (BMI ≥ 40 kg/m^2^) obese adults.

## 2. Methods

### 2.1. Design and Eligibility Criteria

This retrospective cohort study was conducted in France in adults with obesity (BMI ≥ 30 kg/m^2^) admitted for initial obesity assessment in the Clinical Nutrition Department of the University Hospital of Clermont-Ferrand between January 2010 and February 2013. Patients with a history of bariatric or major digestive surgery, genetic or orphan disease, or with a chronic inflammatory disease or an organic health disorder likely to interfere with nutritional status and feeding behavior were excluded. Patients were divided into three groups according to the time of onset of overweight: the CH group (childhood), in which the weight gain started in childhood (before age 11 for females and before age 13 for males); the ADO group (adolescence) for which the weight gain started in adolescence (from age 11 for females and from age 13 for males); the AD group (adult) for which the weight gain started in adulthood (after age 18).

### 2.2. Data Collection

Sociodemographic data, including date of birth and sex, were collected during the hospital admission of patients using identity documents and checked during the interview at the first medical visit. Anthropometric and clinical data included height (m), weight (kg), waist circumference (cm), and arterial pressure (mm/Hg). Height was measured using a SECA mechanical drop meter to within 0.5 cm. Body weight was measured within 0.1 kg in underwear and bare feet using calibrated scales. Waist circumference was measured with a tape measure above the iliac crests. Blood pressure was measured lying down at rest with a Dinamap V100 electronic sphygmomanometer and a cuff designed for obesity. A body shape index (ABSI) was calculated from waist circumference, height, and BMI according to the formula: ABSI (m^11/6^/kg^2/3^) = waist circumference (m)/(BMI (kg/m^2^)^2/3^ × Height (m)^1/2^) [16]. The body composition variables were measured fasting in the morning at rest using a Bodystat^®^ Quadsan 4000 bio-impedance meter. Lean and skeletal muscle mass were calculated with specific formulas [17,18]. Lean and muscle mass were divided by height squared to obtain fat-free mass (FFMI) and skeletal muscle mass (SMI) indices, expressed in kg/m^2^, respectively. The fat-to-lean mass ratio (FyM) was taken as an index of sarcopenia (FyM < 0.4 is normal; FyM = 0.4–0.8 indicates increased fat mass vs. lean mass without lean body mass deficit; FyM > 0.8 indicates sarcopenic obesity) [19].

Medical history was collected at the time of the medical visit. This included a medical history of type 2 diabetes, arterial hypertension, and the nature of the treatments received by the patient.

Overweight history was collected at the time of the dietary consultation. This included weight at age 18 and at the onset of pathological weight gain. These variables were used to calculate BMI at age 18, duration of overweight or obesity, and percentage of weight gain in adulthood compared with weight at age 18 years.

Biological data collected were the standardized assay values of venous blood fasting variables as part of the initial obesity assessment of each patient. The biological analyses were carried out in the biological analysis laboratory of the University Hospital of Clermont-Ferrand. The variables collected were metabolic (fasting glucose, insulinemia, glycated hemoglobin, triglycerides, total cholesterol, and HDL- and LDL-cholesterol), inflammatory (CRP and ferritinemia), and micronutritional (Vitamin D). The HOMA index of insulin resistance was calculated from glucose and insulinemia according to the formula: HOMA-IR = (glucose mmol/L × insulinemia mIU/l)/22.5. Insulin resistance was defined by an index greater than 2.5 [20]. LDL-cholesterol was calculated from the lipid variables of the blood test by the Friedewald formula [21]. Normal Vitamin D values (≥30 µg/L) were distinguished from deficiencies (≤10 to <30 µg/L) and severe deficiencies (<10 µg/L) [22]. Metabolic syndrome was defined according to the NCEP-ATP III criteria (National Cholesterol Education Program Expert Panel 2002) by the presence of at least three of the following five conditions: waist circumference > 102 cm for males or >88 cm for females, blood pressure ≥ 130/85 mmHg or taking antihypertensive therapy, fasting blood glucose ≥ 1.10 g/L or taking anti-diabetic treatment, triglycerides ≥ 1.50 g/L, and HDL-cholesterol < 0.40 g/L for males or <0.50 g/L for females [23].

### 2.3. Statistical Analyses

The study population is described by frequency and percentage for categorical data and by the mean and standard deviation (or median and interquartile range for non-normal distribution) for continuous data.

Metabolic syndrome was analyzed using a Chi-squared test (or Fisher’s exact test when appropriate) for categorical data and the Student’s *t*-test (or the Mann–Whitney test when normality was not reached) for continuous data.

Normality was graphically assessed using the Shapiro–Wilk test.

Analyses of the time of overweight onset were carried out using a Chi-squared test (or Fisher’s exact test when appropriate) for categorical data and analysis of variance (or the Kruskal–Wallis test when normality was not reached) for continuous data.

To take into account potential confounders in the analysis of the relationship between the early onset of overweight and metabolic syndrome (as some patient’s characteristics were found to be associated with both the early onset of overweight and metabolic syndrome), we performed a propensity score (with early onset of overweight as the dependent variable) in order to balance CH, ADO, and AD groups on potential confounders. We then applied an inverse propensity score weighting on the same model as a sensitivity analysis.

The propensity score was computed using a multilevel logistic regression model with the early onset of overweight as the dependent variable, adjusted for age, ABSI, and sex, which were the main potential confounders shown in the univariate analysis [24]. Patient characteristics according to early overweight onset are shown before and after propensity score weighting (Table 1).

### 2.4. Ethical Approval

This study was conducted according to the guidelines laid down in the Declaration of Helsinki. All procedures involving human subjects/patients were approved by the CECIC (Comité d’Ethique des Centres d’Investigation Clinique) Rhône-Alpes-Auvergne, IRB 00005921.

Ethics approval was obtained on 30 June 2017 by the CECIC.

## 3. Results

**Figure 1:**
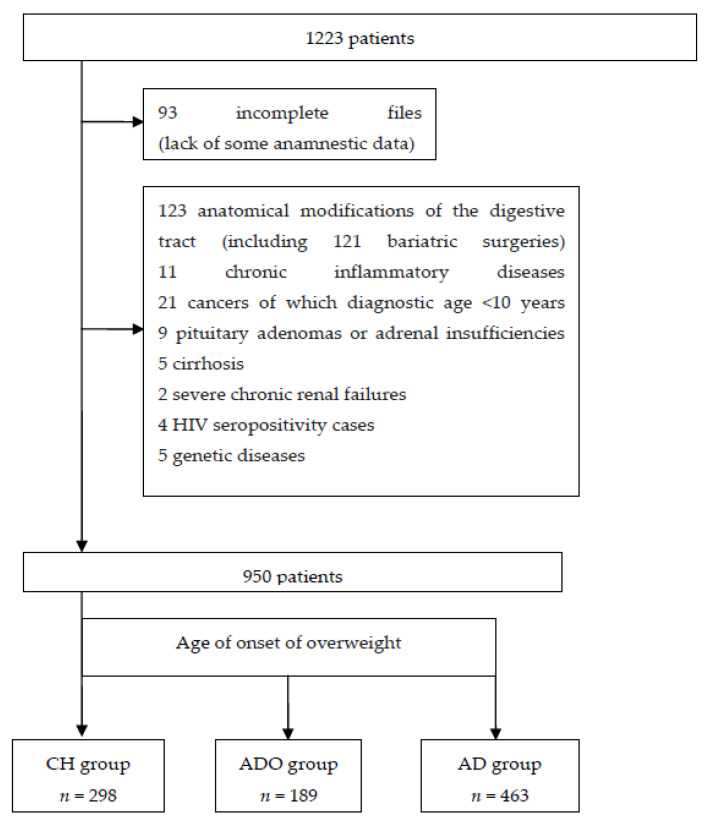
List of patient details.

Of the 1223 obese adult patients hospitalized for an initial obesity checkup, 93 with incomplete medical records and 180 with a previous history of bariatric surgery or chronic inflammatory, genetic, or orphan diseases or cancer with a diagnosis date of fewer than ten years were excluded (Figure 1).

Overall, this study included 950 subjects divided into three groups according to the time of overweight onset: CH (*n* = 298), ADO (*n* = 189), and AD (*n* = 463). The characteristics of the three groups are reported in Table 1.

### 3.1. Anthropometric, Clinical, and Medical History Characteristics

Most of the patients were female, with a significantly higher proportion in the ADO group (86.2 vs. 75.5% CH and 73.9% AD, *p* = 0.002). They were aged 44.3 ± 13.8 years, the AD group being significantly older (51.1 ± 11.8 years vs. 36.5 ± 11.9 in CH and 40.1 ± 12.5 years in ADO, *p* < 0.001).

Severe and morbid obesity accounted for 30.1 and 60.0% of patients, respectively. The mean BMI of the patients was 42.5 ± 7.0 kg/m^2^ and was higher in the CH group (44.4 ± 7.8 kg/m^2^ vs. 42.0 ± 5.7 kg/m^2^ in ADO and 41.6 ± 6.8 kg/m^2^ in AD, *p* < 0.001). All patients had abdominal obesity according to the NCEP ATP III criteria, and excessive waist circumference was highest in the CH group. The prevalence of arterial hypertension, dyslipidemia, or type 2 diabetes according to the NCEP ATP III criteria was not significantly different according to the period of overweight onset after adjusting for the propensity score.

### 3.2. Overweight History

The duration of weight gain was longer in the CH and ADO groups (37.4 ± 16.2 years and 29.2 ± 13.0 years, respectively, vs. 17.7 ± 10.9 years for the AD group, *p* < 0.001) after adjustment for the propensity score. BMI at age 18 years was higher in the CH group (29.8 ± 6.7 kg/m^2^) than in the ADO group (28.6 ± 5.7 kg/m^2^) or the AD group (24.3 ± 4.1 kg/m^2^, *p* < 0.001). The mean weight gain percentage at age 18 years was significantly higher in the AD group (80.8 ± 34.8% vs. 52.6 ± 31.9% in ADO and 47.2 ± 33.5% in CH), *p* < 0.001).

### 3.3. Body Composition

The index scores of the mean lean and skeletal muscle mass were not significantly different in the three groups after adjusting for the propensity score. According to the fat-to-lean mass ratio, the prevalence of sarcopenic obesity was not significantly different across the three groups.

### 3.4. Biological Data

The median fasting blood glucose level of patients in the CH group was lower than that of the ADO and AD groups (fasting blood glucose: 0.96 ± [0.85; 1.04] g/L in CH vs. 0.98 [0.88; 1.15] in ADO and 0.98 [0.87; 1.12] in AD, *p* = 0.01). The prevalence of insulin resistance (HOMA-IR) was not significantly different in the three groups: CH 65.2 vs. ADO 71.5 and AD 77.0, *p* = 0.12). Mean and median lipid values did not differ significantly among the three groups. Median ferritinemia was slightly higher in the ADO and AD groups (105 [45; 213] μg/L in ADO and 116 [56; 229] in AD vs. 86 [55; 169] in CH, *p* = 0.01).

The prevalence of the different age-of-onset variables of overweight presented in Table 1 were comparable by sex stratification.

### 3.5. Metabolic Syndrome and its Criteria

#### Description

The prevalence of metabolic syndrome in our population was 57.8% with a [54.6–61.0] 95% CI and was not significantly different in the three groups (59.8% in AD vs. 54.4% in CH vs. 57.7% in ADO, *p* = 0.59) after adjustment for the propensity score (Table 1). The prevalence of dyslipidemia, arterial hypertension, and type 2 diabetes measured or treated (according to the NCEP ATP III criteria) were not significantly different in the three groups after adjustment.

### 3.6. Univariate Analysis of Metabolic Syndrome by Age, Sex, BMI, Weight History, Body Composition, and Biological Variables

Univariate analysis found that sex and age were associated with metabolic syndrome, with higher prevalence in males and with advancing age (Table 2). Regarding overweight history, BMI at age 18 and weight changes since age 18 were also associated factors, with prevalence increasing with increasing BMI or weight gain. Regarding body composition, the fat mass index was associated with metabolic syndrome, with prevalence increasing as this index increased. The fat-to-lean ratio was not associated with metabolic syndrome, but ABSI was associated with metabolic syndrome. Regarding the biological variables, ferritinemia and Vitamin D were associated with metabolic syndrome, with prevalence increasing as ferritinemia increased and as Vitamin D levels decreased.

### 3.7. Multivariate Analysis of Metabolic Syndrome with Propensity Score Weighting

The association between the presence of metabolic syndrome in adulthood and the onset of being overweight was not statistically significant after weighting on propensity scores of sex, age, and ABSI (Table 3). Logistic regression found weight gain after age 18 is an independent determinant of metabolic syndrome, with the odds ratio increasing with the percentage of weight gain (>50% weight gain: OR = 1.75 [1.07–2.88], *p* = 0.026).

A sensitivity analysis was performed by grouping subjects that became obese during childhood or adolescence vs. adults, using the same methods as the main analysis. Results confirmed that there were no differences in metabolic syndrome prevalence according to the age of obesity onset in either univariate or multivariate analyses.

## 4. Discussion

We have shown in the present study that weight gain in adulthood is an independent determinant of metabolic syndrome [25] despite greater fatness in childhood-originating adult obesity. These results suggest that the development of obesity before adulthood may have a less deleterious effect than gaining body mass in adulthood. However, our study did not show any significant association between the onset of overweight and the later development of metabolic syndrome in adult obese subjects. This observation is consistent with data in the literature that do not show any protective role of childhood obesity in relation to the metabolic complications of obesity in adulthood [13,15].

In our study, weight gain in adulthood by more than 50% of weight at age 18 increased the risk of developing metabolic syndrome. With respect to body composition, lean and skeletal muscle mass indices were not found to be independent determinants of metabolic syndrome [26]. Our results support a predominant role of fat mass in the genesis of metabolic syndrome compared with lean mass. It is not a lean mass deficit that is associated with metabolic syndrome but rather an inflation of the fat mass compared with the lean mass [19]. Regarding biological variables, glycemia and lipid values were not significantly associated with the overweight age of onset after adjustments for the propensity score. As these variables are very significantly correlated with age, the initial differences were probably due to the variance in the average age of the populations. Insulin resistance was positively associated with BMI and sex but not with the age of weight gain onset, suggesting that insulin resistance appeared with obesity regardless of when the latter developed. This is an important finding for prevention and confirms that action against obesity should not be abandoned in adulthood. This result is also consistent with those of Lloyd et al. in all the articles analyzed except for Thearle et al., who found that the action of insulin was negatively correlated with BMI in childhood [27].

Because the mean ages of the patients in the CH and AD groups were significantly different, the propensity score was required to ensure the comparability of the groups. Further studies of a prospective nature, such as studying the localization of adipose tissue and the morphology of adipocytes together with the growth curves in the health records, are now needed to better specify the trajectories and metabolic risks of these patients.

Finally, in future studies on the impact of weight gain onset on late comorbidity occurrence, larger cohorts might explore deeper phenotyping, considering not only the course of BMI but also adipose tissue distribution, cell size, or even metabolic activity of adipocytes as potential predictive indicators of late metabolic complications, as proposed recently by Arner’s group [28].

## 5. Conclusions

Although the early onset of obesity in childhood leads to a greater body mass and higher BMI in later adulthood, the present study found that metabolic syndrome prevalence was no greater, suggesting that it was the magnitude rather than the period of weight gain that aggravated the risk of metabolic syndrome in adulthood. Aside from absolute BMI values, the weight trajectory of patients with obesity provides relevant clinical information for evaluating metabolic risk.

## Figures and Tables

**Table 1:** Description of the characteristics of the study population (*n* = 950) and its three groups: CH (*n* = 298), ADO (*n* = 189), and AD (*n* = 463).

Anthropometric, Clinical, and Anamnestic Characteristics								
	CH ^a^	ADO ^a^	AD ^a^	*p*	CH ^b^	ADO ^b^	AD ^b^	*p*
Female %	75.5	86.2	73.9	0.002	78.8	76.8	76.6	0.85
Age (years)	36.5 (11.9)	40.1 (12.5)	51.1 (11.8)	<0.001	45.5 (14.9)	44.1 (13.6)	45.0 (13.0)	0.73
BMI ^c^ (kg/m^2^), mean (sd)	44.4 (7.8)	42.0 (5.7)	41.6 (6.8)	<0.001	42.6 (7.44)	42.4 (5.84)	42.9 (7.66)	0.76
30–35%	7.4	6.3	13.6	<0.001	15.1	5.4	10.9	0.13
35–40%	23.2	32.8	33.5		26.1	31.8	30.2	
>40%	69.5	60.8	52.9		58.8	62.8	58.9	
Waist size (cm), mean (sd)	129 (17.3)	124 (12.8)	126 (15.0)	0.001	126 (16.9)	126 (13.2)	128 (16.4)	0.55
Treated arterial hypertension (%)	22.6	27.0	48.2	<0.001	38.5	36.7	37.8	0.94
Treated type 2 diabetes (%)	11.9	15.0	21.0	0.003	15.2	21.9	16	0.23
Treated dyslipidemia (%)	10.4	11.3	25.6	<0.001	21.3	16.7	19.6	0.66
Weight history								
Weight at age 18, mean (kg) (sd)	88.9 (22.5)	77.9 (18.0)	63.7 (13.2)	<0.001	80.5 (21.4)	78.1 (18.2)	65.7 (14.1)	<0.001
BMI at age 18, mean (sd)	32.2 (7.5)	28.9 (5.8)	23.6 (3.7)	<0.001	29.8 (6.7)	28.6 (5.7)	24.3 (4.1)	<0.001
Weight gain since age 18(kg), average percentage (sd)	43.3 (34.9)	50.1 (32.9)	79.3 (33.2)	<0.001	47.2 (33.5)	52.6 (31.9)	80.8 (34.8)	<0.001
Duration of weight gain (years), mean (sd)	27.9 (11.5)	25.6 (12.0)	21.4 (11.5)	<0.001	37.4 (16.2)	29.2 (13)	17.7 (10.9)	<0.001
Body composition								
% lean mass, mean (sd)	55.1 (6.0)	55.5 (5.3)	57.5 (6.6)	<0.001	56.6 (6.7)	56.2 (5.7)	56.3 (6.4)	0.93
% fat mass, mean (sd)	44.9 (6.0)	44.4 (5.3)	42.5 (6.6)	<0.001	43.4 (6.7)	43.8 (5.7)	43.7 (6.4)	0.93
FFMI ^d^ (kg/m^2^), mean (sd)	24.1 (3.8)	23.0 (2.5)	23.7 (3.7)	0.007	23.8 (3.7)	23.6 (2.8)	23.9 (4.0)	0.69
SMI ^e^ (kg/m^2^), mean (sd)	11.0 (2.1)	10.3 (1.4)	10.5 (2.3)	0.002	10.6 (2.2)	10.6 (1.7)	10.7 (2.4)	0.82
FyM ^f^ ratio (%)								
<0.4	1.48	0	3.0	0.001	4.1	0	2.1	0.271
0.4–0.8	43.2	48.3	54.8		49.0	51.2	49.5	
>0.8	55.4	51.7	42.2		46.9	48.8	48.4	
ABSI ^g^ (m^11/6^/kg^2/3^), mean (sd)	0.0806 (0.0061)	0.0805 (0.0054)	0.0824 (0.0054)	<0.001	0.0812 (0.0056)	0.0812 (0.0057)	0.0816 (0.0055)	0.54
Biological characteristics								
Fasting glycemia (g/L), median [IQR]	0.94 [0.85; 1.03]	0.96 [0.87; 1.09]	1.00 [0.90; 1.16]	<0.001	0.96 [0.85; 1.04]	0.98 [0.88; 1.15]	0.98 [0.87; 1.12]	0.01
HDL ^h^-cholesterol (g/L), mean (sd)	0.45 (0.14)	0.46 (0.12)	0.47 (0.13)	0.17	0.48 (0.14)	0.47 (0.12)	0.45 (0.12)	0.19
LDL ^i^-cholesterol (g/L), mean (sd)	1.17 (0.35)	1.18 (0.31)	1.20 (0.35)	0.53	1.21 (0.36)	1.17 (0.33)	1.18 (0.34)	0.75
Triglyceridemia (g/L), median [IQR]	1.14 [0.84; 1.64]	1.14 [0.81; 1.66]	1.23 [0.90; 1.67]	0.06	1.13 [0.81; 1.75]	1.14 [0.8; 1.66]	1.23 [0.88; 1.66]	0.66
Hba1c ^j^ median [IQR]	5.70 [5.40; 6.10]	5.70 [5.40; 6.20]	6.00 [5.60; 6.50]	<0.001	5.9 [5.5; 6.1]	5.7 [5.4; 6.6]	5.9 [5.6; 6.3]	0.27
Homa IR ≥ 2.5 (%)	72.3	69.1	75.9	0.23	65.2	71.5	77.0	0.12
CRP (mg/L), median [IQR]	5.90 [3.10; 11.2]	6.40 [3.6; 10.6]	5.70 [3.00; 9.72]	0.12	4.80 [2.90; 9.5]	6.5 [3.50; 10.4]	6.3 [3.0; 10.9]	0.08
Ferritin (µg/L), median [IQR]	82 [42; 161]	79 [40; 179]	126 [59; 247]	<0.001	86 [55; 169]	105 [45; 213]	116 [56; 229]	0.01
Vitamin D (µg/L), mean (sd)	14.1 (7.5)	14.4 (8.0)	13.9 (8.0)	0.80	15.2 (7.81)	14 (7.9)	13.8 (7.76)	0.28
<10	4.3	3.3	4.9	0.59	5.4	3.5	4.1	0.48
10–30	60.3	64.1	56.6		65.2	61.1	57	
>30	35.5	32.7	38.5		29.4	35.5	38.9	
Metabolic syndrome and its criteria								
Measured or treated arterial hypertension ^k^ (%)	56.0	50.3	72.1	<0.001	64.2	59.5	61.2	0.62
Measured or treated type 2 diabetes ^k^ (%)	20.1	25.4	37.4	<0.001	22.7	33.6	32.1	0.06
Hyper HDL ^j^ (%)	62.1	62.8	55.0	0.07	59.7	57.7	61.1	0.80
Hypertriglyceridemia ^k^ (%)	30.9	29.8	32.9	0.70	33.5	32.1	32.5	0.95
Metabolic syndrome (%)	51.7	52.9	63.7	0.001	54.4	57.7	59.8	0.59

^a^: unadjusted; ^b^: after propensity score weighting; ^c^: body mass index; ^d^: fat-free mass index; ^e^: skeletal muscle mass index; ^f^: fat mass/lean mass; ^g^: body shape index; ^h^: high-density lipoprotein; ^i^: low-density lipoprotein; ^j^: glycated hemoglobin; ^k^: according to the NCEP ATP III metabolic syndrome criteria.Inverse propensity score weighting was then applied to analyze the metabolic syndrome with early overweight onset as the main covariate and was adjusted for clinically relevant factors and those highlighted in the univariate analysis. Results are shown as odds ratios with 95% confidence intervals. A sensitivity analysis was performed by grouping CH and ADO vs. AD using the same methodology (computing propensity score as the weighting for the metabolic syndrome analysis). All tests were two-sided, and a *p*-value of <5% was considered significant. All analyses were performed using Stata software (Version 15.0, StataCorp, Texas, TX, USA).

**Table 2:** Univariate analysis of factors associated with metabolic syndrome, using propensity score weighting.

	No MetS (*n* = 401)	MetS (*n* = 549)	*p*
Anthropometric, clinical, and anamnestic characteristics			
Female (%)	83.3	72.1	<0.001
Age, mean (sd)	41.28 (13.83)	46.54 (13.28)	<0.001
BMI, mean (sd)	41.70 (6.86)	43.16 (7.11)	0.002
30–35	13.5	7.8	0.003
35–40	32.2	28.6	
>40	54.4	63.6	
Weight history			
BMI at 18, mean (sd)	28.41 (7.20)	26.79 (6.43)	0.001
Weight gain since age 18 (kg), average % (sd)	53.90 (38.65)	67.38 (35.71)	<0.001
Duration of weight gain (years), mean (sd)	23.34 (11.33)	24.72 (12.30)	0.128
% of lean mass, mean (sd)	56.09 (5.81)	56.59 (6.61)	0.25
% of fat mass, mean (sd)	43.91 (5.81)	43.43 (6.59)	0.27
FFMI (kg/m^2^), mean (sd)	10.31 (1.88)	10.86 (2.27)	<0.001
SMI (kg/m^2^), mean (sd)	0.80 (0.18)	0.79 (0.21)	0.42
FyM, mean (sd)	0.08 (0.01)	0.08 (0.01)	0.99
ABSI (m^11/6^/kg^2/3^), mean (sd)	111.25 (21.28)	118.00 (24.64)	<0.001
Biological characteristics			
CRP, median [IQR]	6 [3; 10]	6 [3.2; 10.6]	0.29
Fasting glycemia (g/L), median [IQR]	0.91 [0.85; 0.98]	1.05 [0.93; 1.24]	<0.001
Hba1c, median [IQR]	5.7 [5.4,;5.9]	6 [5.6; 6.65]	<0.001
Insulinemia, median [IQR]	14.6 [8.8; 20.8]	19.4 [13.2; 27.7]	<0.001
Homa IR, median [IQR]	3.1 [1.8; 4.5]	5 [3.15; 7.95]	<0.001
Homa IR ≥ 2.5			
Ferritin (µg/L), median [IQR]	75 [38; 150]	128 [59; 236]	<0.001
Vitamin D (µg/L), mean (sd)	15.05 (8.01)	13.34 (7.68)	0.003
<10	15 (4.7)	18 (4.1)	0.05
10–30	206 (64)	243 (55.7)	
>30	101 (31.4)	175 (40.1)	

**Table 3:** Multivariate analysis of factors associated with metabolic syndrome using propensity score weighting.

Variables		
	OR [95% CI]	*p*
Onset of overweight		
Childhood (Ref)		
Adolescence	1.14 [0.69; 1.92]	0.60
Adulthood	0.99 [0.62; 1.56]	0.95
Weight gain by more than 50% of weight at age 18 years (%)	1.75 [1.07; 2.88]	0.03
Age < 35 (Ref)		
35–45	1.27 [0.81; 1.98]	0.30
45–55	1.60 [0.97; 2.62]	0.07
>55	2.11 [1.04; 4.26]	0.04
ABSI 1000 (m^11/6^/kg^2/3^)	1.07 [1.04–1.11]	<0.0001
Female	0.60 [0.37; 0.96]	0.03

## Data Availability

Not applicable.
